# Childhood Grandparent Closeness and Midlife Mental Health

**DOI:** 10.1093/geroni/igaf122.1863

**Published:** 2025-12-31

**Authors:** Cheyenne Garcia

**Affiliations:** University of Michigan, Ann Arbor, Michigan, United States

## Abstract

We propose to examine the association between childhood closeness to a grandparent and mid-life depressive symptoms and stress. We used data from the Social Relations study, a longitudinal representative study of individuals in Detroit. We collected Wave 1 data in 1992 and follow-up data in 2005 (Wave 2) and 2015 (Wave 3). We examined closeness to a grandparent in childhood and later depressive symptoms, self-esteem, self-efficacy, and stress in 205 children (51.7% girls, 61.6% white). We defined closeness as being within the grandchild’s closest 10 social relationships. The median age (SD) was 10.4 years old (1.4) in Wave 1, 23.3 (1.5) in Wave 2, and 33.4 (1.5) in Wave 3. In Wave 1, 77 children (37%) were close to at least one grandparent. After controlling for age and sex, childhood closeness to a grandparent was almost significantly associated with lower depressive symptoms in Wave 3 (F(1,109)=3.90, p = 0.05), but not at all in Wave 2(F(1, 140) = 0.88, p = 0.35). We found that self-efficacy mediated much of this relationship, such that grandparent closeness in childhood was associated with higher self-efficacy in Wave 2 and higher self-efficacy was associated with greater depressive symptoms in Wave 3. Children who were close to their grandparent were bothered by fewer hassles in Wave 2 (13% vs. 18%). Closeness to a grandparent influences self-efficacy, which mediates the relationship with depressive symptoms and, possibly, stress. Promoting grandparent involvement in childcare could benefit children’s mental health in midlife.

